# Use of bioengineered human commensal gut bacteria‐derived microvesicles for mucosal plague vaccine delivery and immunization

**DOI:** 10.1111/cei.13301

**Published:** 2019-04-15

**Authors:** A. L. Carvalho, A. Miquel‐Clopés, U. Wegmann, E. Jones, R. Stentz, A. Telatin, N. J. Walker, W. A. Butcher, P. J. Brown, S. Holmes, M. J. Dennis, E. D. Williamson, S. G. P. Funnell, M. Stock, S. R. Carding

**Affiliations:** ^1^ Gut Microbes and Health Research Programme Quadram Institute Bioscience Norwich UK; ^2^ Defence Science and Technology Laboratory Porton Salisbury UK; ^3^ Public Health England, Porton Porton Salisbury UK; ^4^ Plant Biotechnology Ltd Norwich UK; ^5^ Norwich Medical School University East Anglia Norwich UK; ^6^ Present address: Udo Wegmann, School of Chemistry University East Anglia Norwich UK

**Keywords:** antibodies, gut bacteria, humoral immunity, mucosal vaccine, non‐human primates, outer membrane vesicles, plague

## Abstract

Plague caused by the Gram‐negative bacterium, *Yersinia pestis*, is still endemic in parts of the world today. Protection against pneumonic plague is essential to prevent the development and spread of epidemics. Despite this, there are currently no licensed plague vaccines in the western world. Here we describe the means of delivering biologically active plague vaccine antigens directly to mucosal sites of plague infection using highly stable microvesicles (outer membrane vesicles; OMVs) that are naturally produced by the abundant and harmless human commensal gut bacterium *Bacteroides thetaiotaomicron* (Bt). Bt was engineered to express major plague protective antigens in its OMVs, specifically Fraction 1 (F1) in the outer membrane and LcrV (V antigen) in the lumen, for targeted delivery to the gastrointestinal (GI) and respiratory tracts in a non‐human primate (NHP) host. Our key findings were that Bt OMVs stably expresses F1 and V plague antigens, particularly the V antigen, in the correct, immunogenic form. When delivered intranasally V‐OMVs elicited substantive and specific immune and antibody responses, both in the serum [immunoglobulin (Ig)G] and in the upper and lower respiratory tract (IgA); this included the generation of serum antibodies able to kill plague bacteria. Our results also showed that Bt OMV‐based vaccines had many desirable characteristics, including: biosafety and an absence of any adverse effects, pathology or gross alteration of resident microbial communities (microbiotas); high stability and thermo‐tolerance; needle‐free delivery; intrinsic adjuvanticity; the ability to stimulate both humoral and cell‐mediated immune responses; and targeting of primary sites of plague infection.

## Introduction

Plague is caused by the Gram‐negative bacterium, *Yersinia pestis*. It is an ancient disease, accounting for many deaths over hundreds of years, and still exists in parts of the world today. To protect against infection vaccines must be able to elicit both humoural immunity with neutralizing antibodies and cell‐mediated immunity that is effective at primary mucosal sites of infection 1, 2.

There are currently no licensed plague vaccines in the western world. In the past, heat‐killed whole‐cell vaccines (listed in the US Pharmacopeia) were available and provided protection against bubonic, but not pneumonic plague. However, due to unacceptable reactogenicity these vaccines were discontinued 3. Live‐attenuated vaccines have been used in countries of the former Soviet Union and China although, due to unacceptable reactogenicity and the risk of reversion to full virulence, they have not been licensed for use elsewhere, including the United States 4.

Fraction 1 (F1) and LcrV (virulence; V antigen) *Y. pestis* proteins encoded by the Fra/pMT1 and pCD1 plasmids, respectively 5, have been identified as the major protective antigens responsible for preventing phagocytosis of *Y. pestis* (F1) and regulating type III secretion (V antigen) 6. The present emphasis on developing F1‐ and V antigen‐based vaccines is on recombinant protein‐based subunit vaccines (rF1V) that incorporate chemical adjuvants. If a *Y. pestis* variant occurs with a mutation in the V antigen then these F1‐V vaccines may not provide protection via immunity to the V component; however, immunity to the F1 component could still provide some limited protection 7, 8. In addition, the duration of protection against pneumonic infection provided by these as a subunit vaccine, in which the F1 and V antigens are presented as a fusion protein, is also uncertain 9. Furthermore, the requirement for injection by needle to deliver these, and other current vaccines, is problematical. It is associated with: risks of cross‐contamination; lack of patient compliance; the high cost of mass immunization; and a requirement for cold chain delivery and storage. Importantly, injected vaccines provide only partial or no protection at the primary mucosal site of plague infection 2, 10. Collectively, these issues constrain the use of existing plague vaccines, particularly in resource‐poor low‐income settings, and these restrictions are reflected in the World Health Organization (WHO)’s draft therapeutic product profile 11.

The use of nanoparticle‐based platforms is a new approach to the development of more effective mucosal vaccines against pathogens such as those that cause the plague. These include virus‐like particles, immune stimulating complexes, polymeric nanoparticles, inorganic nanoparticles, liposomes and emulsions – all of which have the potential to overcome the high production costs and safety concerns associated with live vaccines and the weak immunogenicity and adjuvanticity issues associated with subunit and recombinant protein‐based vaccines 10. These nanoscale carrier technologies enable conformationally correct antigens to be incorporated into highly stable nanoparticles. This allows for control over the spatial and temporal presentation of antigens to the immune system, leading to their targeted and sustained release. An overlooked component of platform nanoscale vaccines are bacterial microvesicles, and in particular, outer membrane vesicles (OMVs) of Gram‐negative bacteria 12. While many synthetic nanoparticles are capable of transferring heterologous antigens to antigen‐presenting cells (APC), the ability to efficiently stimulate the immune system is often not inherent 13. However, OMVs can combine high stability with antigen presentation and native adjuvanticity, making them an attractive vaccine platform for further development 14.

OMV production by vesiculation is a fundamental characteristic of Gram‐negative bacteria that is unrelated to bacterial lysis or membrane instability that fulfils the key requirements of a prokaryotic secretion process 15. Nanoscale OMV proteoliposomes that contain immunogenic components derived from the bacterial outer membrane and periplasm are capable of targeting APCs [including dendritic cells (DC)] 16, 17, 18, leading to T and B cell immunity. Recent research on OMVs from pathogenic bacteria, including *Neisseria meningitidis* and *Vibrio cholerae*, supports the case for their being good vaccine candidates 19; OMVs derived from *N. meningitidis* have been used safely and effectively as vaccine platforms for the control of serogroup B meningococcal (MenB) disease outbreaks 20, 21. OMV‐based vaccines offer significant advantages over conventional vaccines because they are: non‐replicating; provide needle‐free delivery; target mucosal sites; have an established safety record; can elicit innate and antigen‐specific adaptive immune responses; and possess self‐adjuvant properties [i.e. microbe associated molecular pattern molecules (MAMPs) such as lipopolysaccharide (LPS)]. The current limitations of pathogen‐derived, OMV vaccines are: the potential for unintended toxicity due to associated toxins; low expression levels of protective antigens; variable efficacy depending on source and formulation; the need for exogenous adjuvants; and only incomplete protection because of strain variation. In principle, these limitations could be overcome by using OMVs from non‐pathogenic commensal bacteria, engineered to improve their application as vaccines. In support of this, it has recently been shown that OMVs produced by the common human commensal gut bacterium, *Bacteroides thetaiotaomicron* (Bt), are able to access and influence the host’s intestinal epithelial and immune cells 22, 23. This identifies a means by which commensal gut bacteria can influence host cell physiology.

Here we describe the development of a novel vaccine delivery technology, based on engineering Bt, to express the V and F1 antigens of *Y. pestis* in their OMVs for targeted delivery to the gastrointestinal (GI) and respiratory tracts in a non‐human primate (NHP) host. Our findings demonstrate that OMV‐based plague vaccines are an effective means of eliciting both mucosal and systemic antibody responses and systemic cell‐mediated responses. Delivery of OMV vaccines via the respiratory route was particularly effective at eliciting production of antigen‐specific IgG antibodies that were protective in two independent surrogate assays.

## Materials and methods

### Bacteria, media, growth conditions and transformations

Strains of *Escherichia coli* were grown in Luria–Bertani medium at 37°C. Bt strain VPI‐5482 and derivative strains were grown under anaerobic conditions at 37°C in brain heart infusion (BHI) medium (Oxoid, Basingstoke, UK) supplemented with 0·001% haemin (BHIH) or with 0·00005% haemin for OMV preparations. Antibiotics were added as selective agents when appropriate: ampicillin 200 μg/ml and erythromycin 5 μg/ml. The *E. coli* strain J53/R751 was supplemented with trimethoprim 200 μg/ml when grown for 18 h. *E. coli* GC10 was transformed by electroporation using a Gene Pulser II (Bio‐Rad, Watford, UK). Plasmids were mobilized from *E. coli* into Bt following a triparental filter mating protocol 24 using the helper strain J53/R751. The *Y. pestis* strain CO92 (biovar Orientalis, NR641; BEI Repositories) was supplied by the Biodefence and Emerging Infections (BEI) Research Repository (Bethesda, MD, USA) in accordance with International Export and Import Regulatory Requirements and used with kind permission from National Institute of Allergy and Infectious Diseases (NIAID). The organism was stored and handled in accordance with US Biological Select Agent or Toxin requirements and was grown using the conditions and methods described previously 25.

### *Construction of *Y. pestis* F1‐ and V1‐antigen expression vectors*

A synthetic gene construct of 1043 base pairs (bp) encoding the V antigen and a synthetic operon construct of 3826 bp encoding *caf1M*,* caf1A* and *caf1* genes of the *caf1* operon that generates the F1 protein were N‐terminally fused to the OmpA signal peptide of Bt. This created *in‐silico* constructs with codon usages optimized for expression in the same species. Signal peptide prediction was obtained by SignalP at http://www.cbs.dtu.dk/services/SignalP/. During the design of the synthetic constructs the unique *Bacteroides* ribosomal binding site 26 required for efficient expression in *Bacteroides* was accounted for. The resulting gene cassettes for V and F1 were obtained through gene synthesis and subsequently cloned into the *E. coli* plasmids pEX‐A2 and pEX‐K4 (Eurofins, Hamburg, Germany), respectively. The cassettes contained *Bsp*HI and *Eco*RI restriction sites at their 5′ and 3′ ends, respectively, allowing for the translational fusion of the encoded gene to the start codon in the *Bacteroides* expression vector pGH090 26. The genes encoding V1 or F1 were excised from the pEX derivatives using *Bsp*HI and *Eco*RI and ligated into the *Nco*I/*Eco*RI‐restricted pGH090 expression vector, resulting in pGH179 and pGH180, respectively. Finally, the sequence integrity of the cloned fragments was verified through sequencing.

### OMV isolation and characterization

OMVs were isolated following a method adapted from Stentz *et al*. 27. Briefly, cultures of Bt (500 ml) were centrifuged at 5500 ***g*** for 45 min at 4°C and the supernatants filtered through polyethersulphone (PES) membranes (0·22 μm pore‐size) (Sartorius, Göttingen, Germany) to remove debris and cells. Supernatants were concentrated by ultrafiltration (100 kDa molecular weight cut‐off, Vivaspin 50R; Sartorius), the retentate was rinsed once with 500 ml of phosphate‐buffered saline (PBS) (pH 7.4) and concentrated to 1 ml (approx. 700 µg/ml total protein). The final OMV suspensions were filter‐sterilized (0·22 µm pore size). The protein content of the final OMV suspensions was determined using the Bio‐Rad Protein Assay.

The distribution of heterologous proteins within Bt OMVs was established in a proteinase K accessibility/protection assay 27. Briefly, a suspension of 250 μg of OMVs in 0·1 M phosphate/1 mM ethylenediamine tetraacetic acid (EDTA) buffer (pH 7.0) was incubated for 1 h at 37°C in the presence of 100 mg/l proteinase K (Sigma‐Aldrich, Poole, UK). Proteinase K activity was stopped by addition of 1 mM phenylmethanesulphonyl fluoride (PMSF) and samples analysed by immunoblotting.

### Nanoparticle analysis

Videos were generated using a Nanosight nanoparticle instrument (NanoSight Ltd, Malvern, PA, USA) to count OMV numbers in each OMV sample. The mean squared displacement (X) was measured simultaneously for each OMV tracked. The particle diffusion coefficient (D_t_), and hence sphere equivalent hydrodynamic radius (r_h_) were determined using the Stokes–Einstein equation,

### $D_{t}=\frac{k_{B}T}{6\pi \eta r_{h}}$ ,

where k_B_ is Boltzmann’s constant, T is temperature and η is solvent viscosity.

### Immunoblotting

OMV‐V extracts were added to sodium dodecyl sulphate–polyacrylamide gel electrophoresis (SDS‐PAGE) loading buffer (NuPage; Invitrogen, Carlsbad, CA, USA) containing dithiothreitol (Invitrogen). Approximately 7 μg of OMV‐V was loaded onto 12% precast Tris–glycine gels (Novex/ThermoFisher Scientific, Fremont, CA, USA) and separated by electrophoresis at 180 volts for 40 min. Gels were transferred onto a polyvinylidene difluoride membrane at 25 volts over 2 h in a solution containing Tris–glycine transfer buffer (Novex). The membrane was blocked with 10% bovine serum albumin (BSA) in Tris‐buffered saline (TBS) (50 mM Tris‐HCl, 150 mM NaCl, pH 7.5)‐Tween (0·05%) for 30 min at 20^°^C. Blocking solution was then discarded and the membrane incubated for 16 h at 4°C in a 1 : 1000 dilution of a primary mouse anti‐V antibody (Defence Science and Technology Laboratory, Porton Down, UK) in TBS‐Tween with 5% BSA. After washing with TBS‐Tween three times, membranes were incubated for 1 h at 20^°^C in 5% BSA in TBS‐Tween with a 1 : 1000 dilution of horseradish peroxidase (HRP)‐conjugated goat anti‐rabbit IgG (ThermoFisher). After three washes in TBS‐Tween, SuperSignal West Pico chemiluminescent Substrate (ThermoFisher) was used to detect bound antibody.

The F1 content of F1‐OMV formulations was determined using a dot‐blot assay in which serial twofold dilutions of extracts of F1‐OMVs (10 μl) were spotted onto nitrocellulose membranes and air‐dried. Serial dilutions of recombinant F1 protein (rF1) and extracts of native OMVs were also applied to the membranes. The membranes were then processed as described for immunoblotting as above, using a rabbit anti‐F1 anti‐sera (Defence Science and Technology Laboratory) and a secondary HRP‐conjugated goat anti‐rabbit IgG (ThermoFisher). Localization of F1 in Bt OMV preparations was determined by liquid chromatography and mass spectrometry (LC‐MS)‐based proteomics (Proteomics Facility, University of Bristol, Bristol, UK).

### Cytokine analysis

Frozen PBMCs obtained from whole blood by density gradient centrifugation over Ficoll‐Paque Plus (Amersham Biosciences, Chalfont St Giles, UK) were thawed, washed twice with RPMI media containing 10% FBS, 2 mM glutamine and 100 U/ml penicillin/streptomycin and adjusted to a concentration of 5 × 10^5^ cells/ml. Aliquots of 10^6^ cells were plated into individual U wells of 96‐well plates and incubated in triplicate with media alone or media containing 15 μg/ml rV protein for 72 h at 37^°^C in an incubator with 5% CO_2_. Control cultures contained media only. Conditioned media was then harvested, and cytokine content determined using a bead‐based multiplex assay and BD LSRFortessa flow cytometer (BD Biosciences, San Jose, CA, USA), according to the manufacturer’s instructions (BioLegend, San Diego, CA, USA).

### Antibody enzyme‐linked immunosorbent assay (ELISA)

Nunc‐Immuno Microwell 96‐well plates (ThermoScientific) were coated with 15 μg/ml of *Y. pestis* V (BG032/VDJPE1) or F (BG032/FD5Pst2) recombinant proteins (Defence Science and Technology Laboratory) in ELISA coating buffer (0·1 M NaHCO_3_) and incubated for 16 h at 4°C. After washing three times with ELISA wash buffer (PBS with 1 : 2000 Tween‐20), plates were blocked for 3 h with blocking buffer (PBS with 2% BSA) at 20^°^C with gentle agitation. Serum, saliva, salivary gland and bronchoalveolar lavage (BAL) samples were added in dilutions ranging from 1 to 163 840 and the plates were incubated for 16 h at 4°C. After six washes with ELISA wash buffer, 50 μl of 1 : 10 000 goat anti‐monkey immunoglobulin (Ig)G peroxidase conjugated (Sigma; A2054‐1ml) or 1 : 10 000 goat anti‐monkey IgA–HRP antibody (Sigma; SAB3700759) were added and plates were incubated for 1 h at 20^°^C with gentle agitation. Plates were then washed six times with ELISA wash buffer and incubated with 100 μl of TMB high sensitivity substrate solution (BioLegend, San Diego, CA, USA) for 20 min at 20^°^C in darkness. The reaction was stopped by adding 50 μl of 2 N H_2_SO_4_. The plates were analysed in a microplate reader at Abs_450 nm_. Absolute amounts of IgG and IgA antibodies in serum and mucosal samples, respectively, were determined using a modified ELISA incorporating a range of concentrations of purified monkey IgG (Bio Rad) and IgA (Life Diagnostics, Inc., West Chester, PA, USA) and the protocol and reagents described above to generate standard curves from which IgG and IgA concentrations of individual animals were determined.

### Bactericidal assay

This prototype bactericidal assay (BCA) was developed in a series of experiments which optimized incubation times, using a standard anti‐rF + rV anti‐serum generated in cynomolgus macaques. All activities associated with the BCA assay were performed at ACDP3 level containment within a BSL3 safety cabinet. *Y. pestis *CO92, originally sourced by Public Health England (PHE) from the BEI research repository (catalogue number: NR‐641) was used to make a working stock. Working stock was propagated on Columbia blood (COH) agar for 2 days at 26^°^C. A bacterial suspension was then created by harvesting the bacteria from the surface of the agar and subsequent resuspension in TSB. Sterile TSB broth was then inoculated with sufficient bacterial suspension to result in an OD_600nm_ of 0·1. After 3 h incubation at 26^°^C, the mid‐log culture was diluted to an expected *Y. pestis* concentration of 3·3 × 10^3^ colony‐forming units (CFU)/ml ready for use as the inoculum for the bactericidal assay. This process was used in all protocols.

Heat‐treated (to deplete individual sample complement) primate sera were incubated with live *Y. pestis* bacteria in the presence of 25% v/v rabbit complement (Pellfreeze Biologicals, Roger, AR, USA) for 1 h with orbital agitation on a 96‐well plate shaker. After this incubation, the mixture was plated onto COH agar and permitted to be absorbed into the agar at 37^°^C. In preliminary experiments, human volunteer and baby rabbit complement (Pellfreeze Biologicals) were found to be similar in sensitivity to antibody‐directed bactericidal activity against *Y. pestis* CO92. After this incubation, the mixture was plated onto COH agar and permitted to be absorbed into the agar. The COH plates were then incubated at 37°C for 2 days before manual enumeration to determine relative viability. Incubation at 37°C only began once the antibody, complement and bacterial mixture was created in the assay plate. This protocol simulated the natural infection process that would take place once a flea has bitten a mammalian host. No‐antibody, complement‐only controls were included in all assays.

The number of bacteria counted for each test serum anti‐serum was used to calculate the degree of reduction in counts compared with the plate spread with the no‐antibody complement control. The dose of test serum [expressed as percentage (v/v) of serum] required to kill 50% of live *Y. pestis* CO92 was calculated by interpolation of the dose response curve for each sample. Each assessment was conducted in duplicate. Bactericidal antibody assessment assays were conducted at PHE on samples taken on each of the 4 sample days with serum pools from each of the 12 vaccine groups. In all studies, the reference standard anti‐rV plus anti‐rF1 primate serum was also assessed as a means of comparison and measure of reproducibility. In all studies, this standard anti‐serum killed *Y. pestis* in a reproducible manner. All controls (including the no‐antibody, complement‐only no antibody control) were assessed and performed as expected.

### Competitive ELISA

A competitive ELISA (CE‐ELISA) assay was used (as previously described 1) to detect antibodies in immune sera from V‐OMV immunized animals that were able to compete with a monoclonal antibody (mAb 7·3) for binding to recombinant V protein. Briefly, rV antigen was coated onto 96‐well microtitre plates (Dynex, Lincoln, UK) at 5 μg/m1 in 0·05 ml PBS (16–18 h, 4^°^C). After washing in PBS with 0·02% Tween 20, plates were blocked with 0·2 ml 5% w/v skimmed milk powder in PBS (37^°^C, 1 h). After further washing, 0·05 ml mAb 7·3 (1 : 32000 in 1% w/v skimmed milk powder in PBS) was added to each well (equivalent to 80 ng/well) and plates were incubated at 4^°^C for 16 h. Normal, non‐immune NHP serum, also diluted to 1 : 32 000, was added to negative control wells (0·05 ml per well). Plates were then washed prior to adding the test serum at a dilution of 1 : 10 in 1% w/v skimmed milk powder in PBS. A positive control was included comprising a reference serum created by pooling equal aliquots of sera from four macaques previously parenterally immunized with rF1 + rV antigens and prior to surviving challenge with *Y. pestis*. Test and control serum samples were assayed in duplicate. Plates were incubated (1 h, 37^°^C) prior to washing and addition of HRP‐goat anti‐mouse IgG (Serotec, Kidlington, UK; 1 : 2000 in PBS) followed by incubation (37^°^C, 1 h). Plates were washed prior to addition of ABTS substrate (Sigma) with subsequent reading of absorbance at 414 nm. The optical diameter (OD)_414nm_ determined for each test and the reference serum was adjusted by subtraction of the OD_414nm_ determined for the appropriate control serum. The data were calculated from a titration curve for loss of binding of the mouse antibody, with increased concentration of human serum.

### Ethics statement

All animals used in these studies were cynomolgus macaques (*Macaca fasciculuaris*) obtained from the characterized breeding colonies managed by PHE that have been established and maintained as closed colonies for more than 20 years. Animals were housed in compatible social groups, in accordance with the Home Office (UK) Code of Practice for the Housing and Care of Animals Bred, Supplied or Used for Scientific Purposes, December 2014, and the National Committee for Refinement, Reduction and Replacement (NC3Rs), Guidelines on Primate Accommodation, Care and Use, August 2006. All animals were aged between 3 and 5 years and none of the animals had been used previously for experimental procedures, were free of herpes B‐virus, tuberculosis (TB), simian immunodeficiency virus (SIV) and simian T‐cell leukemia virus (STLV) and were inspected by the named veterinary surgeon prior to entry into the study. All animal procedures and study design were approved by the Public Health England, Porton Down Animal Welfare and Ethical Review Committee, and authorized under an appropriate UK Home Office project licence (30/2993 and P76404B45).

### Generation of standard NHP reference anti‐sera

In order to enable development of a functional biological assay of relevance to these studies, anti‐sera to F1 and V were generated by immunizing two cynomolgus macaques with either recombinant F1 (batch BG032\FD5Pst2; Defence Science and Technology Laboratory) or recombinant V (batch BG032\VDJPE1; Defence Science and Technology Laboratory) antigens produced from *E. coli* [1] and formulated into 20% (v/v) alhydrogel adjuvant. Both vaccines were prepared and supplied to PHE by Defence Science and Technology Laboratory. Two primates (one male, one female) were immunized with either vaccine on two occasions 3 weeks apart. The dose on each vaccination occasion was 50 μg injected intramuscularly in a 250 μl volume. Once an immune response was confirmed to have been induced via ELISA, subjects were anaesthetized by intramuscular injection of ketamine (15 mg/kg) and blood samples were collected from the heart prior to euthanasia by intracardiac injection of a lethal dose of anaesthetic (Dolelethal, 140mg/kg; Vétoquinol UK Ltd, Towcester, UK) in order to generate the reference stock anti‐sera 56 days after primary immunization. The sera were collected after centrifugation of serum separation tubes which had permitted the blood to clot. Serum aliquots were created and frozen at below –20°C until required. Stool samples were also collected for reference purposes into OMNIgene GUT OMR‐200 tubes.

### OMV vaccines and vaccination

The immune status for each NHP was assessed with regard to *Y. pestis* antigens at 14 and 0 days before immunization. For OMV‐V/F1 vaccine studies, animals were randomly allocated to groups of four (two males and two females per group) and were immunized with OMV‐V or OMV‐F1 vaccine formulations containing 12·5, 25 or 50 μg of V or F1 protein via the intranasal route or with 50 μg of V/F1 protein via the oral route in a total volume of 1 ml PBS. Nasal dosing of vaccine was conducted using a stepwise droplet method to alternate nares using a sterile flexible plastic tube (13GA plastic feeding tube; Instech Laboratories Inc., Plymouth Meeting, PA, USA) and oral administration was performed using a flexible dosing tube to ensure accurate delivery. Twenty‐eight days later, all NHPs received a booster immunization via the same route of administration. Blood, stool, saliva and rectal and nasal swab samples were collected at days –14, 0, 28, 42 and at the study end‐point of 56 days with body weight, temperature, axillary and inguinal lymph node scores and haemoglobin concentration determined at each sampling time‐point. Animals were sedated by intramuscular (i.m.) injection with ketamine hydrochloride (Ketaset, 100 mg/ml; Fort Dodge Animal Health Ltd, Southampton, UK; 10 mg/kg) for simple procedures such as blood sampling and vaccination that required removal from their housing. At the end of the study, animals were sedated with ketamine (15 mg/kg, i.m. administration), weighed and clinical data collected. Prior to euthanasia, anaesthesia was deepened using medetomidine hydrochloride (Sedator, 1 mg/ml; Dechra Veterinary Products, Shrewsbury, UK) 50 mg/kg and a BAL was performed. BAL fluid samples were collected using a bronchoscope (Allscope XE30 4‐mm flexible bronchoscope; VES, Rochford, UK). BAL consisted of three consecutive washes performed, each using 20‐ml volumes of Hanks’ balanced salts (Sigma‐Aldrich, Poole, UK), which was instilled in the lungs and collected. Exsanguination of each animal was affected via the heart before termination by the injection of a lethal dose of anaesthetic (140 mg/kg) (Dolethal; Vétoquinol UK Ltd). At necropsy, lung, spleen, liver, heart, lymph nodes, kidney, brain, stomach and intestine were collected for histopathology and routinely processed. The tissues were stained with haematoxylin and eosin (H&E) and examined by light microscopy and evaluated subjectively by a pathologist, blinded to the treatments and groups in order to prevent any bias.

### Statistical analysis

CE‐ELISA data were analysed using Graph Pad Prism software version 6 and expressed as mean ± standard error of the mean (s.e.m.). Statistical comparisons were made using one‐way analysis of variance (anova) or unpaired *t*‐test. The survival data were expressed as Kaplan–Meier survival curves and statistical significance was determined by log‐rank test. *P* < 0·05 was considered statistically significant. Cytokine multiplex data were analysed using one‐way anova with Bonferroni’s multiple comparison post‐test to compare PBS and rV values for each cytokine.

## Results

### Study rationale

The aim of this immunogenicity and reactogenicity study was to determine the suitability of OMVs produced by the human commensal gut bacteria, Bt, as a delivery platform for plague vaccine antigens that could activate mucosal and systemic immune responses in an NHP host, and be capable of protecting against plague infection. Mice are a common experimental model system used in preclinical studies of human drugs and vaccines. However, the NHP model is better suited to answering key questions about Bt OMV vaccine efficacy and safety in humans. This is because the genetic relatedness of primates, and hence greater physiological and microbiological similarity to humans, requires less interpolation compared to other animal model systems 28, 29, 30, 31. Therefore, the use of NHPs de‐risks the development pathway for Bt‐OMV vaccine by providing assurance that the NHP microbiome and histological integrity of the GI tract, and other associated tissues, are not adversely affected following immunization. In addition, the use of NHP anti‐sera as a surrogate to quantify bactericidal activity and protection against infection will help to pave the way for assessment of the protective effect of Bt OMV vaccination in humans.

### *Expression of* Y. pestis* vaccine antigens in Bt OMVs*

**Figure 1:**
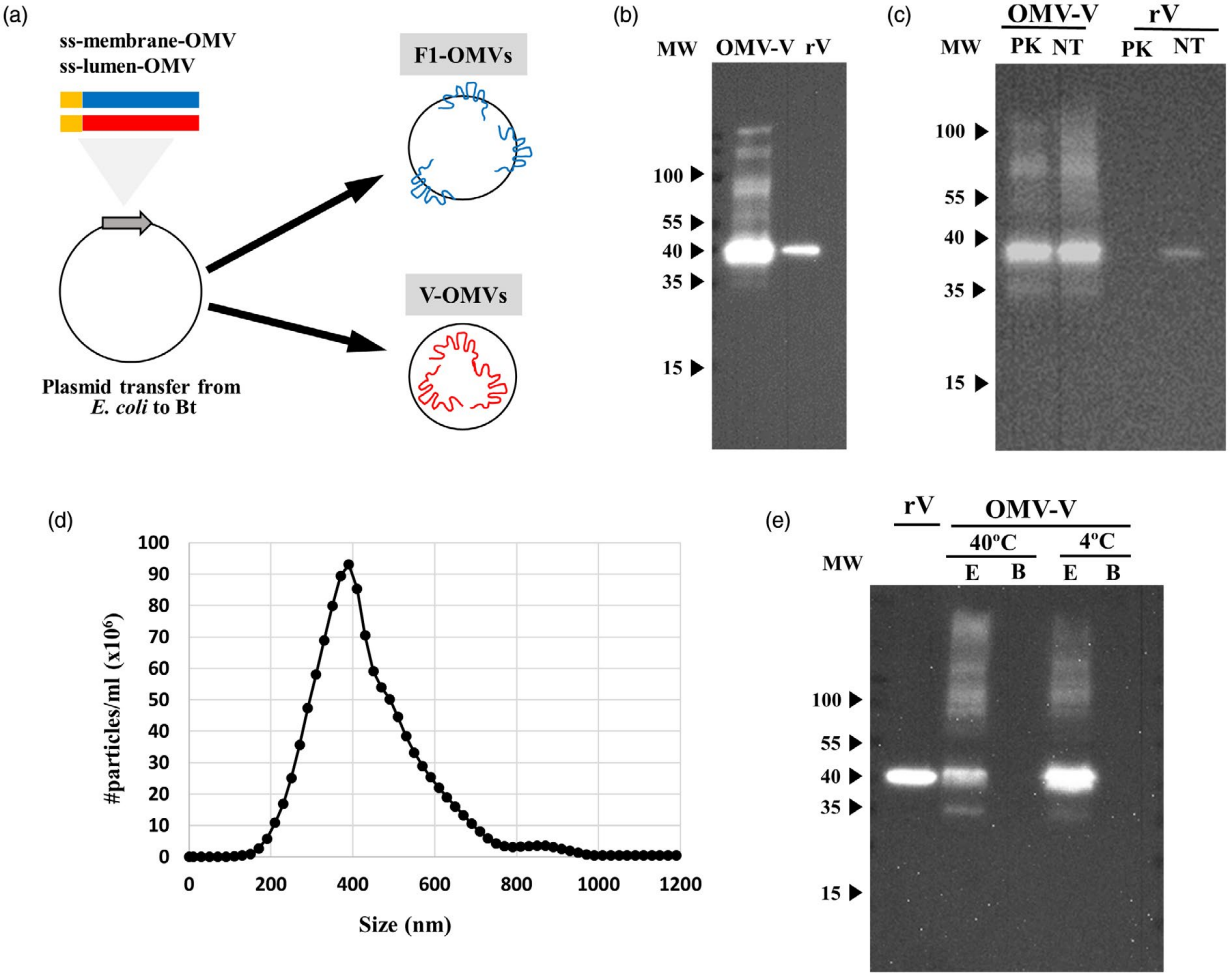
*Bacteroides thetaiotaomicron* (Bt) outer membrane vesicle (OMV) plague vaccines. (a) Schematic of cloning procedure for the expression of *Yersinia* virulence proteins Fraction 1 (F1) and V antigen (V) at the surface or in the lumen, respectively, of Bt OMVs. The Bt secretion signal sequence is indicated in yellow and is fused at the N‐terminus of the F1 and V genes. (b) Expression of V antigen in OMV lysates determined by immunoblotting (IB). (c) Determination of protein location after treatment with proteinase K (PK). IB of V antigen with and without pretreatment of V‐OMVs or recombinant V (rV) with proteinase K. NT = not treated. (d) Size distribution of Bt V‐OMVs by nanoparticle tracking analysis. The OMV suspension was diluted 100 times. (e) Thermostability of V‐OMVs assessed by storing preparations at different temperatures for 8 weeks prior to analysing OMV extracts (E) or storage buffer (B) for presence of V antigen by IB. Molecular weight (MW) is expressed in kDa.

It is possible to engineer Bt to target protein antigens to a specific location in OMVs. The antigen can be either secreted as a soluble protein in the lumen of OMVs by making a fusion with an appropriate secretory signal peptide or, alternatively, it can be targeted to the surface of OMVs by making fusions of the antigen with OMV surface proteins. Accordingly, genes encoding the V and F1 *Y. pestis* proteins were successfully cloned downstream of sequences encoding the N‐terminal signal peptides of the major OMV protein OmpA (BT_3852), the products of which were contained within the lumen or outer membrane of OMVs (Fig. 1a). The constructs were generated in *E. coli* hosts and then mobilized into Bt via a triple‐filter mating protocol using a helper strain. Immunoblotting of whole cell and OMV lysates of recombinant Bt strains confirmed expression of the V antigen (approximately 15 μg/ml of total protein) (Fig. 1b). A dot‐blot assay was used to detect F1 protein in OMV lysates (approximately 10 μg/ml of total protein) with its presence being confirmed by LC‐MS proteomics of OMV lysates (data not shown). The luminal *versus* outer membrane distribution of the proteins in Bt OMVs was established using a protease protection assay. This assay demonstrated that V protein distribution was within the lumen of OMVs (Fig. 1c). The presence of F1 protein in extracts of F1‐OMV was confirmed by LC‐MS proteomics, with its absence in F1‐OMV samples pretreated with proteinase K being consistent with its localization to the OMV outer membrane. V and F1 containing OMVs had an average size of ~400 nm (Fig. 1d) and exhibited high thermostability with minimal loss of vaccine antigen content after storage for 6 weeks at either 4^°^C or 40^°^C (Fig. 1e and data not shown).

### OMV immunization

OMV vaccination followed a prime and a single boost dosing regimen as depicted in Supporting information, Fig. S1, with each animal serving as their own control and reference for evaluating OMV vaccine responses. Levels of antigen‐specific antibodies were assessed in serum samples obtained at two pre‐immunization time‐points (days –14 and 0) and at three time‐points post‐immunization (days 28, 42 and 56). Oral and nasal administration are the preferred routes of vaccination to generate protective immunity at primary sites of plague infection 9. To identify which of these routes was optimal for Bt OMV plague vaccines we measured both local and systemic antigen‐specific V and F1 IgA and IgG antibodies following delivery. For oral delivery we used a dose of 50 μg of V antigen formulated in Bt OMVs, which is mid‐range of the vaccine dose used previously in cynomolgus macaques 32, and is equivalent to that required for a human dose 7. In considering the potential risks of administering agents via the intranasal route and its accessibility to the systemic circulation and the brain, we used a range of OMV vaccine antigen doses (12·5, 25 and 50 μg) to assess safety and tolerability and determined the lowest dose required to induce a strong immune response.

### Host response to F1‐OMV plague vaccines

**Figure 2:**
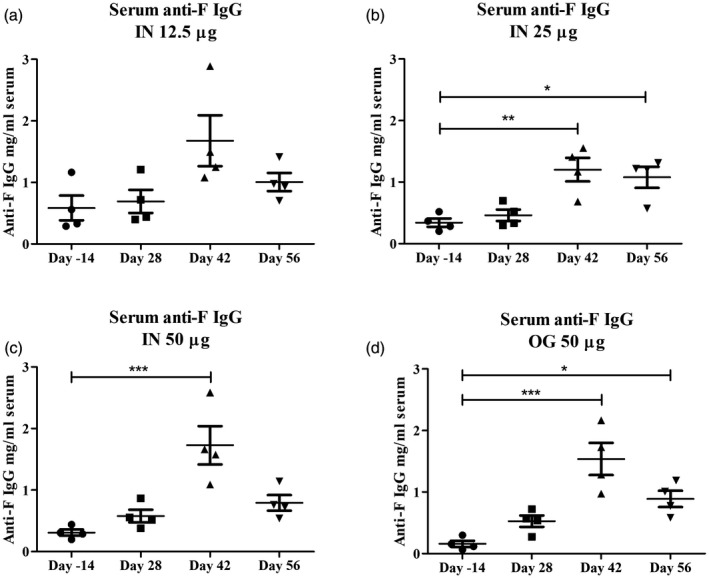
Humoral systemic immune response to Fraction 1‐outer membrane vesicles (F1‐OMV) vaccines. The quantity and titre of F‐antigen‐specific immunoglobulin (Ig)G in the sera of animals immunized with F‐OMVs via the intranasal or oral route was determined prior to immunization (day –14 ) and at three time‐points post‐immunization (days 28, 42 and 56) by enzyme‐linked immunosorbent assay (ELISA) using an initial serum dilution of 1 : 10 prior to serial one in four dilutions. Data expressed as mean ± standard error of the mean (s.e.m.). *< 0·05: **< 0·01; ***< 0·001.

F1‐OMV vaccine formulations were evaluated by measuring antigen‐specific IgA levels in mucosal secretions and tissues and antigen‐specific IgG levels in the serum (Fig. 2 and Supporting information, Fig. S2). F1‐OMVs generated antigen‐specific IgG serum antibodies (~0·5–1·5 μg/ml) after both oral and intranasal immunization at day 42 and then decreased (~0·5 μg/ml) at day 56. The fact that the antibody levels did not increase until day 42 reflects the importance of and requirement for a booster immunization (at day 28). There was no clear evidence for an effect of antigen dose on the levels of F1 antibodies produced, as similar levels of F1‐specific IgG were recorded in animals receiving 12·5, 25 or 50 μg of F1‐OMVs (Fig. 2a). There was also no clear evidence for the superiority of oral *versus* nasal delivery of F1‐OMVs in terms of levels of antigen‐specific IgG antibodies generated (Fig. 2a). By comparison with serum IgG antigen‐specific responses, F1‐OMVs elicited weak mucosal immune responses with low levels of antigen‐specific IgA present in saliva (Supporting information, Fig. 2a–d) and BAL (Supporting information, Fig. S2e) samples, irrespective of the route of administration. It was not possible to detect F1‐specific IgA antibodies in the salivary glands of F1‐OMV immunized animals (Supporting information, Fig. S2f).

### Host response to V‐OMV plague vaccines

**Figure 3:**
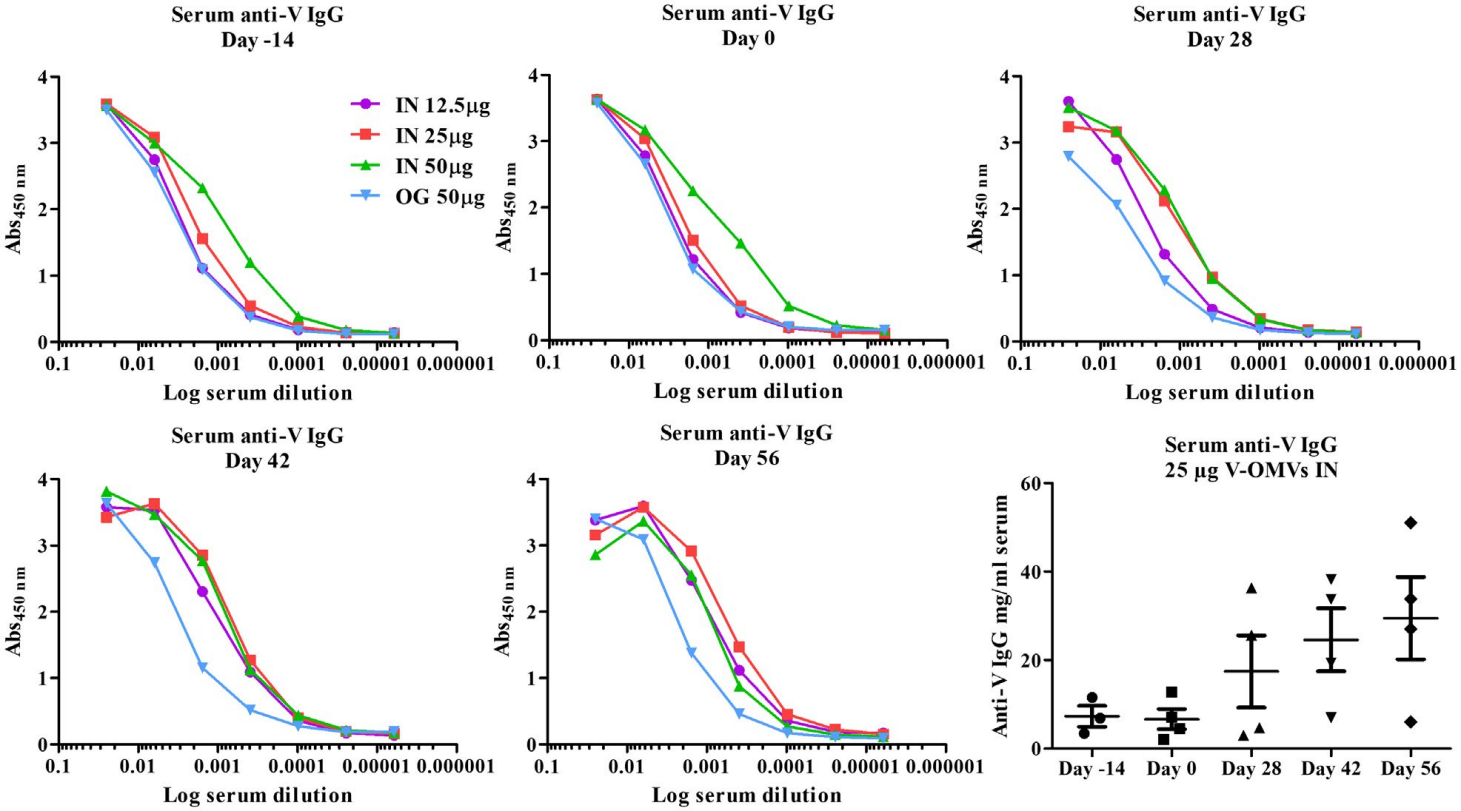
Humoral systemic immune response to V antigen‐outer membrane vesicles (V‐OMV) vaccines. The quantity and titre of V‐antigen specific immunoglobulin (Ig)G in the sera of animals immunized with V‐OMVs via the intranasal or oral route was determined prior to immunization (days –14 and 0) and at three different time‐points post‐immunization (days 28, 42 and 56) by enzyme‐linked immunosorbent assay (ELISA) using an initial serum dilution of 1 : 10 prior to serial one in four dilutions. Each line graph represents the mean value for each group (*n* = 4). The levels of V‐specific IgG in individual animals immunized with 25 μg of V‐OMVs via the intranasal route is shown in the bottom right graph with the horizontal lines in each category representing the mean ± standard error of the mean (s.e.m.).

**Figure 4:**
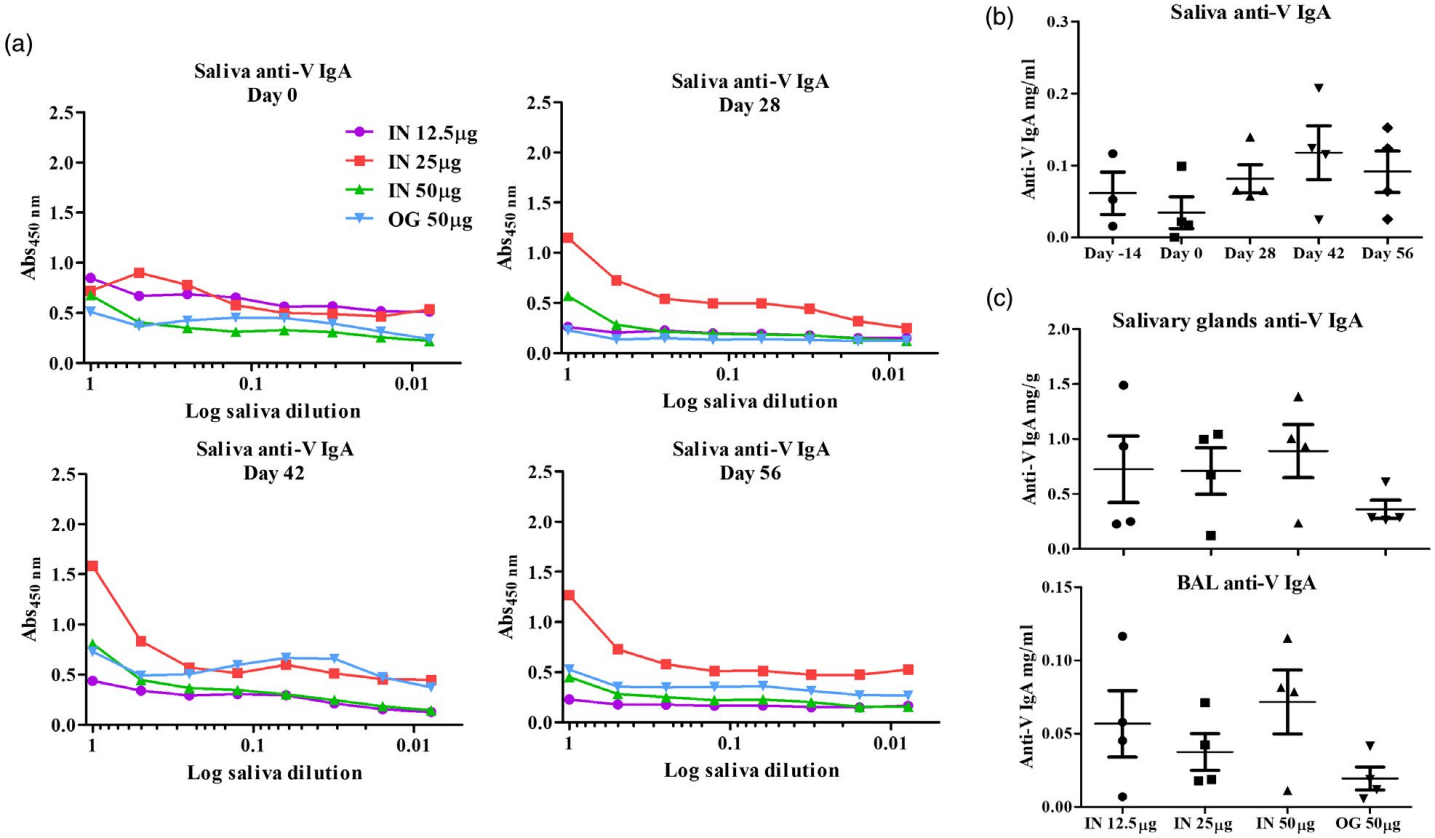
Mucosal humoral immune response to V antigen‐outer membrane vesicles (V‐OMV) vaccines. (a) The quantity and titre of V‐antigen specific immunoglobulin (Ig)A in the saliva of animals immunized with V‐OMVs via the intranasal or oral route was determined prior to immunization (day 0) and at three different time‐points post‐immunization (days 28, 42 and 56) by enzyme‐linked immunosorbent assay (ELISA) using serial one in two dilutions. Each line graph represents the mean value for each group (*n* = 4). (b) Levels of V‐specific IgA in saliva samples of individual animals immunized with 25 μg of V‐OMVs via the intranasal route. The horizontal lines in each category represent the mean ± standard error of the mean (s.e.m.). (c) Levels of V‐specific IgA in salivary glands and bronchoalveolar lavage (BAL) samples for animals immunized intranasally or orally with V‐OMVs analysed at day 56 post‐immunization.

V‐OMV vaccines generated strong antibody responses systemically and at mucosal sites (Figs. 3 and 4). Analysis of serum anti‐V‐specific IgG antibodies showed that the intranasal immunization route for V‐OMV immunization generated higher titres of antibodies than oral immunization (Fig. 3); in contrast, for F1‐OMVs there were no differences in IgG responses between the two vaccine delivery routes (Fig. 2). The highest titres of V‐specific IgG at the study end‐point were in animals intranasally vaccinated with 25 μg of V‐OMV, with antibody levels increasing over the study period (Fig. 3). In mucosal samples, low titres of V‐specific IgA were recorded in the saliva at all time points analysed (Fig. 4a), with the highest titres being seen at day 42 (Fig. 4b). Consistent with the superior performance of intranasally delivered V‐OMVs for generating V‐specific IgG antibodies, higher levels of V‐specific IgA were recorded in the saliva of animals immunized intranasally compared to those immunized via the oral route (Fig. 4a,b). The 25‐μg dose of intranasally administered V‐OMVs achieved the highest titres of V‐specific IgA in saliva (Fig. 4a), and were similar to serum V‐specific IgG antibody responses (Fig. 3). By comparison, salivary gland V‐specific IgA antibody titres at day 56 were similar in animals immunized with 12·5, 25 or 50 μg of antigen (Fig. 4c). The titre of V‐specific IgA antibodies in the BAL were lower than in either the saliva or the salivary glands, with no evidence of vaccine dose‐level‐dependent responses, as each dose of V‐OMVs elicited similarly low levels of V‐specific IgA (Fig. 4c).

**Figure 5:**
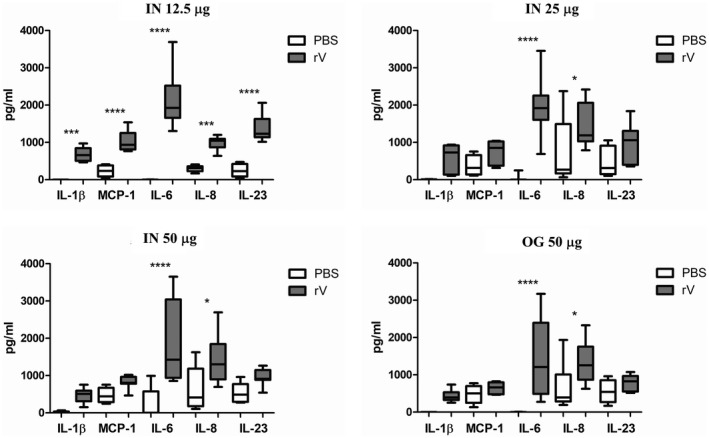
Cytokine production by peripheral blood mononuclear cells (PBMCs) from V antigen‐outer membrane vesicles (V‐OMV)‐immunized animals. PBMCs obtained from animals previously immunized with V‐OMVs via the intranasal (i.n.) or oral route at day 56 post‐immunization were cultured in the presence or absence [phosphate‐buffered saline (PBS)] of recombinant V protein (rV) for 72 h, after which time supernatants were analysed for cytokine content using a multiplex bead assay and flow cytometry. Limit of detection for given values was interleukin (IL)‐1β < 1·11 pg/ml, MCP‐1 < 1·61 pg/ml, IL‐6 < 1·45 pg/ml, IL‐8 < 1·02 pg/ml, IL‐23 < 1·44 pg/ml. Given values represent the group mean ± standard error of the mean (s.e.m.) of duplicate samples, *n* = 4 per group. One‐way analysis of variance (anova) with Bonferroni’s multiple comparison post‐test was used to compare PBS and rV values for each cytokine; *P ≤ 0·05, *** P ≤ 0·001, **** P ≤ 0·0001.

As an indicator of cell‐mediated immune responses to OMV vaccines, we analysed the recall response of peripheral blood lymphocytes from animals immunized intranasally or orally with V‐OMVs after restimulation with rV antigen *in vitro*. PBMCs from V‐OMV immunized animals constitutively produced varying levels of monocyte chemoattractant protein (MCP)‐1, IL‐6, IL‐8 and/or IL‐23 during culture in complete media alone (Fig. 5). In the presence of rV, significant increases in the levels of IL‐6 (*P* < 0·0001) were seen in all PBMC samples irrespective of the immunization route or the dose of V‐OMVs used. Significant increases in IL‐1β (*P* < 0·001), MCP‐1 (*P* < 0·0001), IL‐8 (*P* < 0·001) and IL‐23 *P* < 0·0001) were also seen in rV‐stimulated PBMC samples from animals immunized intranasally with the lowest dose of V‐OMV (12·5 μg). At higher doses of intranasal or oral administered V‐OMVs IL‐8 was the only cytokine, in addition to IL‐6, that was secreted at levels significantly higher (*P* < 0·05) after rV stimulation compared to control cultures. Other cytokines included in the analysis that were not detected in any PBMC sample or were present at levels below the detection limit of the assay (≤ 1·0 pg/ml) included IFN‐γ, TNF‐α, IL‐10, IL‐12p70, IL‐17A and IL‐18 (data not shown).

### Reactogenicity of V‐OMV vaccines

**Figure 6:**
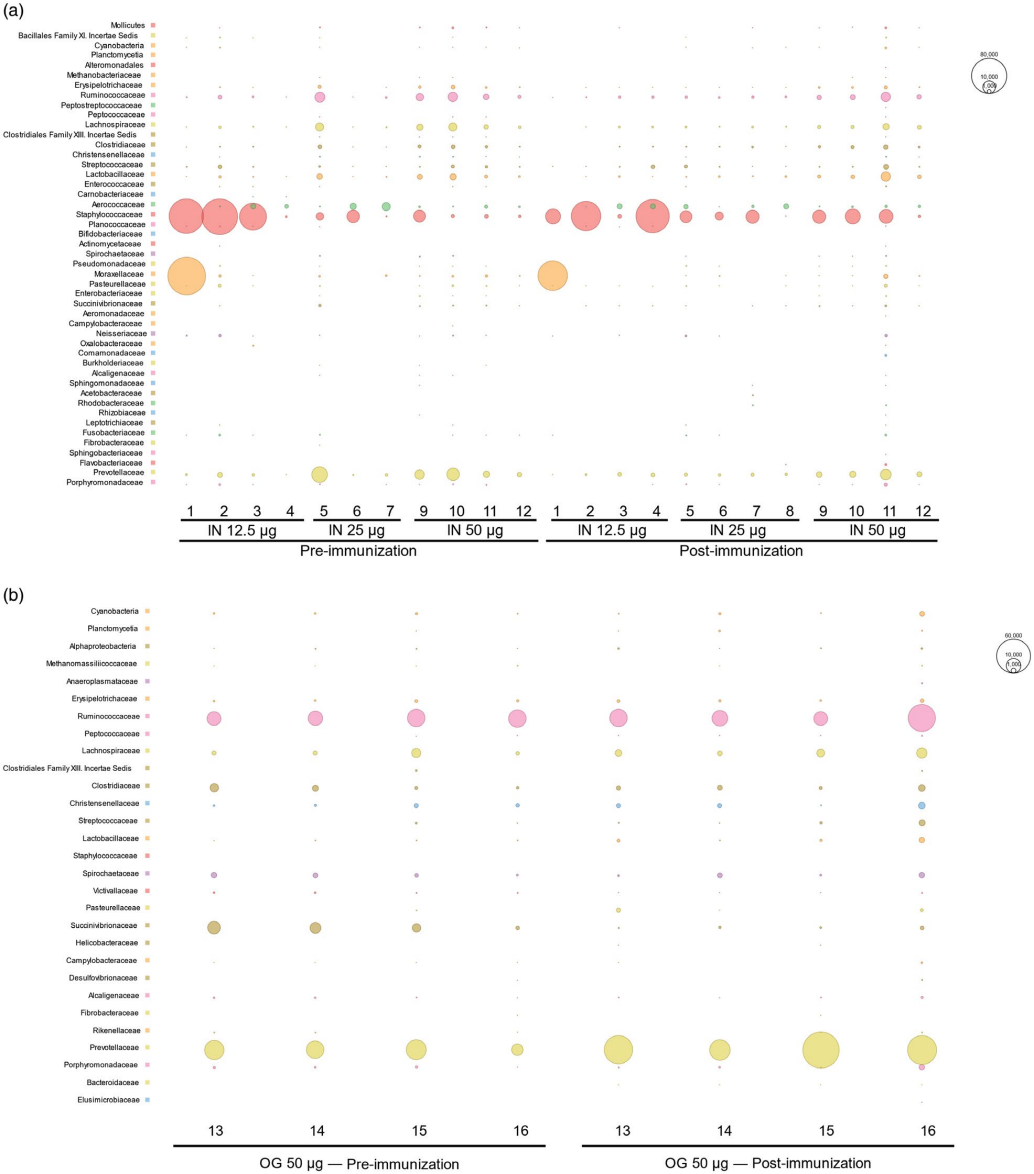
Bubble charts constructed using MEGAN Community Edition for 16S rRNA sequences in which the radius of each bubble is proportional to the number of 16S rRNA sequences obtained from animals immunized with V antigen‐outer membrane vesicles (V‐OMV) to visualize the degree of relatedness between samples. Numbers 1–16 represent individual animals. (a) Nasal microbiota of animals pre‐ and post‐immunization with different vaccine doses via the intranasal route. (b) Faecal microbiota of animals pre‐ and post‐immunization with V‐OMVs via the oral route.

Biosafety of F‐OMV and V‐OMV vaccines were based on histopathology of tissue routinely recovered at necropsy (Supporting information, Fig. S3), and profiling of the resident microbial populations (microbiotas) of the GI and respiratory tracts using 16S rRNA sequence‐based community profiling of faecal‐ and nasal swab‐derived DNA samples taken pre‐ and post‐OMV immunization (Fig. 6). Independent blinded evaluation of various tissues (lung, spleen, liver, heart, lymph nodes, kidney, brain and regions of the GI tract) at necropsy revealed no macroscopic signs of pathogenic infection or pathology.

Evidence of recent immune activation were seen in the lymphoid tissues of the spleen and lymph nodes in a proportion of animals in each group receiving F1‐OMV immunization; this comprised the presence of scattered secondary follicles with mitotic figures and apoptotic cells in the splenic white pulp (Supporting information, Fig. S3a) and cortex of the lymph nodes (Supporting information, Fig. S3b). Microscopic examination of tissues from animals receiving the highest dose of V‐OMVs (50 μg) identified regions of organized and enlarged lymphoid structures and follicles within the spleen and lungs (Supporting information, Fig. S3f) in the absence of any infection or bacteria. Lymphoplasmacytic cell infiltrates were observed with some frequency in the mucosa of all parts of the GI tract (Supporting information, Fig. S3c–e); this is a common finding in our experience with macaques, and their presence probably reflects a low‐grade, chronic–active gastritis/enteritis/colitis that may or may not have been associated with clinical signs such as diarrhoea.

16S rRNA community profiling and bubble chart analysis of sequence data revealed that there was considerable interindividual variation in both the nasal (Fig. 6a) and faecal (Fig. 6b) microbiotas of NHPs at baseline. Post‐immunization with V‐OMVs via the intranasal routes did not noticeably alter the profiles of nasal microbiota at any vaccine dose (Fig. 6a). In faecal microbiota the bacterial composition did not significantly change, although small changes in relative abundance were noted (Fig. 6b). For example, the average prevalence of *Prevotellaceae* increased from 30% at pre‐immunization to 44% after immunization. By contrast, there were decreases in the average prevalence of *Succinivibrionaceae* (from 9 to 0·4%). These findings are consistent with V‐OMVs having a minor impact on resident prokaryotic communities in the upper respiratory tract.

### Functionality of OMV‐elicited IgG antibodies

Two independent assays were used to assess the functionality of immune sera from OMV immunized animals and to determine their usefulness as potential immune correlates for protection in humans. The first assay used was a CE‐ELISA 1 that quantified the ability of immune IgG to compete for binding to the *Y. pestis* V antigen. This assay utilizes a monoclonal antibody (mAb 7·3) which has previously been shown to protect mice fully by passive transfer against direct exposure to *Y. pestis* 33. Alongside a passive transfer assay, we have previously demonstrated that the competitive ELISA provides a potential *in‐vitro* correlate of protection for plague 1. The second assay was a prototype BCA specifically developed for this study, which assessed the level of antibody‐mediated complement killing of *Y. pestis* in serum samples using the *Y. pestis* reference strain CO92 as the target.

**Figure 7:**
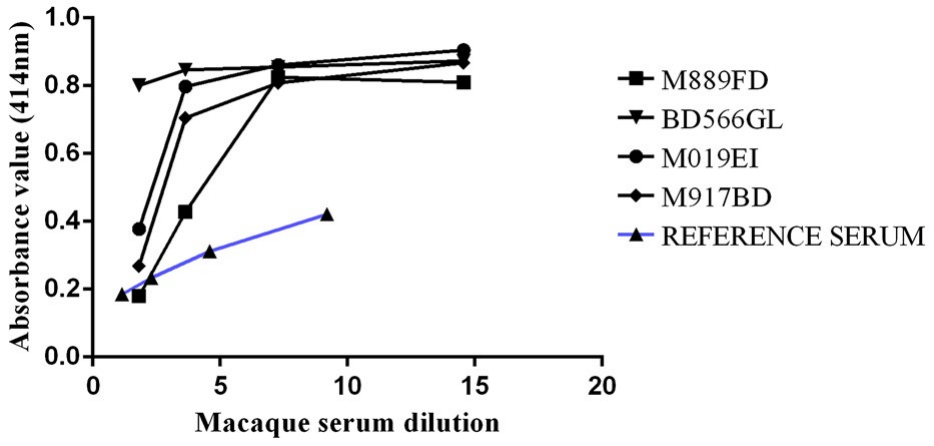
A competitive enzyme‐linked immunosorbent assay (ELISA) assay to assess the functionality of immune sera from V antigen‐outer membrane vesicles (V‐OMV)‐immunized animals. Serial dilutions of sera from animals immunized with V‐OMVs identified by individual code numbers shown in the key were tested for their ability to displace a monoclonal V antibody from the surface of the V antigen immobilized in microtitre plate wells. The level of competing activity of immune non‐human primate (NHP) serum was determined after subtraction of the activity of non‐immune serum. Reference immune serum was from NHPs immunized intramuscularly with recombinant Fraction 1 (F1) and V antigen plus adjuvant.

Serum samples collected at the study end‐point from representative animals within each of the different routes of immunization and dose‐level groups were assayed for their ability to displace mAb 7·3 from binding to rV *in vitro*. The data are presented as a titration line for loss of binding of the mouse monoclonal antibody with increasing concentrations of test samples (see Materials and methods) using, as a reference, macaque immune sera obtained by parenteral immunization with rF1+ rV proteins (Fig. 7). Values were also corrected for any non‐specific activity by subtracting values obtained using normal, non‐immune, sera. The sera from animals intranasally immunized with V‐OMVs at all doses inhibited, to some extent, the binding of mAb 7·3, which at the higher serum concentrations were comparable to the activity of the reference sera. Sera from animals immunized with 25 or 50 μg of V‐OMVs had the highest titres of competitive antibodies (Fig. 7). In contrast, sera from animals immunized orally with V‐OMVs had low levels, or no antibodies, capable of competing with mAb 7·3 for binding to V antigen.

The BCA provided a functional activity assessment of the different test groups of samples taken from the immunized NHPs. Throughout the assays conducted, the reference anti‐sera (generated by immunizing macaques with recombinant rF1 and rV) provided consistent dose–response BCA behaviour (Table 1).

**Table 1:** Summary of serum antibody bactericidal assay outputs (ED_50_ in units of % serum†)

Vaccine	Dose (μg)	Route	Day 0	Day 28	Day 42	Day 56
OMV‐F1	50	i.n.	12·1	36·2	14·9	>45
OMV‐V	50	i.n.	0·6	5·5	0·8	14·9
OMV‐F1	25	i.n.	1·4	1·6	1·5	6·2
OMV‐V	25	i.n.	1·9	0·5	15·3	13·7
OMV‐F1	12.5	i.n.	>45	21·6	>45	39·2
OMV‐V	12.5	i.n.	>45	>45	20·9	22·5
OMV‐F1	50	OG	>45	17·0	13·0	> 45
OMV‐V	50	OG	>45	1·8	6·3	>45
rF1+rV Alhydrogel	50	i.n.	–	–	–	10·7*

OG = orogastric administration; i.n. = intranasal administration; i.m. = intramuscular administration; OMV = outer membrane vesicles; OMV‐V = *Bt* OMVs containing *Y. pestis* V antigen; OMV‐F1 = *Bt* OMVs containing *Y. pestis* Fraction 1 (F1) antigen; r = recombinant protein produced in *Escherichia coli*; ED_50_ = 50% effective dose.

* Average of six determinations; – = not included in the study design.

^†^ the initial dilution of antibody in the assay was 45%, hence the limit of the assay was nominally set at 45%. Sera found to have an ED_50_ below the limit of detection were assigned an ED_50_ of > 45%.

There was, however, a high background BCA reactivity in many of the macaques at day 0 which was not specific to a particular housing group. Assessment of the health records of the study subjects did not uncover any unreported health conditions and no veterinary adverse health observations were made. Although screened and found to be clear of all known high‐consequence pathogens in primates, a routine rectal swab was able to identify *P. aeurugenosa* in one study participant. Although this organism was not causing disease or inducing any observable clinical sign of illness, it is possible that natural background immunity to such a commensal could cross‐react with the type three secretion system (TTSS) components of *Y. pestis*. Conservation of the TTSS has been discussed and demonstrated in other studies 34, 35, 36, 37. Thus, it is postulated that the high background BCA observed in some study animals is due to immunity to TTSS elements of commensal bacteria present in the colony. This hypothesis was further supported by ELISA, which confirmed that there was pre‐existing immunity in some primates which cross‐reacted with *Y. pestis* recombinant V antigen at day 0 (Fig. 3). This immunity was also confirmed in the CE‐ELISA (Fig. 7).

Intranasal administration appeared to result in higher serum bactericidal responses for both OMV‐antigens. All groups immunized with V‐OMVs showed bactericidal activity during the study, with the best response seen at day 42 post‐immunization in the group immunized with 50 μg OMV‐V intranasally. For F1‐OMVs the data also suggested that an intranasal immunization dose of 25 μg was optimal. While day 56 BCA data (Table 1) suggested that there was some waning of immune functional activity in some groups, at day 42 almost all groups (except the lowest intranasally dosed F1‐OMV group) appeared to demonstrate functional immunity to *Y. pestis*.

## Discussion

Using bacterial OMVs generated by the bioengineering of the major human commensal bacterium, Bt, we have successfully developed formulations of plague vaccine antigens suitable for direct delivery to mucosal sites including the respiratory tract, the site of pneumonic plague infection. Bt OMVs incorporating the V antigen generated robust humoral and cell‐mediated immune responses in both the upper and lower respiratory tracts, and/or in the systemic circulation. Using two independent surrogate assays to measure levels of protection, V‐OMV elicited properties in the sera that were important in immune protection, including the ability to kill *Y. pestis*.

Protection against pneumonic plague is essential to prevent epidemic spread. An outbreak in Madagascar in 2017 resulted in more than 2400 confirmed cases of plague (confirmed, probable and suspected) and more than 200 deaths; the majority (~77%) of reported cases were clinically classified as pneumonic plague 38. Foremost among the virulence factors secreted by *Y. pestis* are the F1 and V proteins that are pivotal in preventing phagocytosis (F1) and regulating type III secretion (V) by *Y. pestis*. When secreted by *Y. pestis*, V and other *Yersinia* outer proteins (Yops), also play roles in inhibiting cytokine production, platelet aggregation and apoptosis of macrophages in addition to immune suppression 39. When combined as purified recombinant proteins, V and F1 represent a powerful candidate vaccine that is amenable to alternative formulations other than typical liquid suspension with alum 40 or alhydrogel 3; this allows for mucosal or dual route 41 delivery.

Using a prime and single boost oral or nasal immunization protocol, V‐OMVs effectively induced antigen‐specific IgA in mucosal sites and IgG in the blood; intranasal delivery was the most effective route of administration, particularly for the induction of mucosal IgA responses. Intranasally delivered V‐OMVs were also able to elicit cell‐mediated immune responses, as evidenced by strong recall responses of PBMCs from immunized animals and the production of proinflammatory cytokines. The weaker immunogenicity of V‐OMVs delivered via the oral route may reflect the more hostile environment of the GI tract compared with the nasal mucosa, and the need to overcome significant physical (mechanical digestion), chemical (acidic/alkaline pH), biological (enzymes) and microbiological (the microbiota) barriers prior to accessing inductive immune sites in the lower GI tract. However, despite these obstacles, orally delivered V‐OMVs were still effective in generating functional antibodies, albeit at lower titres than that in animals immunized nasally with V‐OMVs. At the earliest time‐point of serological analysis (28 days post‐immunization), systemic and mucosal antibody responses were established and increased over time. In comparison with recombinant protein‐based vaccines 41, OMV vaccine formulations may, therefore, be less effective at inducing rapid‐onset immune responses (i.e. within 14 days). However, it is possible that more intensive boosting using multiple doses could be used to accelerate the onset of immunity and increase the strength and duration of immune responses (i.e. within 14 days); this is feasible for OMV vaccines that are non‐invasive and more user‐friendly than injected recombinant protein vaccines for which repeated injections would be a problem. Formulations of *N. meningitidis*‐based OMV vaccine formulations (MenBvac, VA‐Mengoc‐BC, PorA P1.6‐24 and MeNZB) rely on a three‐ or four‐dose immunization regimen to provide effective protection in children and adults and control of outbreaks of MenB disease 42. The option to increase the concentration of V antigen in OMV vaccine formulations is not supported by our data; in terms of generating high tires of both mucosal and systemic antibodies intermediate dose of antigen (25 μg) delivered intranasally performed as well as, if not better than, a twofold higher dose.

The assessment of host immune responses in NHPs was complicated by a level of pre‐existing immunity in some individuals prior to OMV immunization. This ‘background’ immunity was confirmed in three independent serum antibody assays conducted at three different laboratories and sites. The health records of the study subjects in question did not show any health conditions and no veterinary health observations were made. More extensive investigations identified that *Pseudomonas aeruginosa* was present in some sample swabs (S.F., unpublished observations). This, however, was not accompanied by symptomatic infection and no veterinary interventions were required. Antibodies to the TTSS and the V antigen of *Y. pestis* are known to cross‐react with that of other pathogenic Gram‐negative bacteria, including *P. aeruginosa*, *Vibrio* species and *Aeromonas* species that encode homologues of the *Yersinia* V antigen 34. While the presence of these bacterial species could not be confirmed in samples collected during the study, it is possible that they had infected these animals at some time prior to this study and were subsequently eliminated by the immune response they invoked; it is also possible that V‐OMV vaccines may have been assisted by such pre‐existing cross‐reactive immunity. It is noteworthy that this phenomenon of ‘background’ immunity was not seen in every animal.

An important immune correlate for protection by a candidate vaccine is the ability to generate neutralizing antibodies that inhibit the killing of host target cells by bacteria and/or are cytotoxic and can actively kill the bacteria 3. We used two assays to demonstrate the generation of neutralizing antibodies in OMV‐immunized animals. The first was a competitive ELISA in which immune sera from V‐OMV immunized animals was able to compete for binding to the protective epitope in the V antigen with a monoclonal antibody (mAb 7·3), which has previously been shown to protect mice fully by passive transfer against direct exposure to *Y. pestis* 1. The second assay is a novel *Y. pestis* BCA developed specifically for this study, in which immune sera from F1‐ and V‐OMV immunized NHPs were shown to kill bacteria via antibody‐dependent complement‐mediated killing (ADCC). Intranasally administered V‐OMVs were particularly effective at generating high titres of bactericidal antibodies. Interpretation of the BCA data is complicated by the suggestion of pre‐existing cross‐reactive immunity in some of the macaques which existed before immunization. This is evident in Fig. 3, where the group which eventually received intranasal vaccination with 50 μg of OMV‐V vaccine had detectable anti‐V immunity 14 days before vaccination and at day 0. This does not, however, account for all the prevaccination BCA activity. Future studies involving the use of mutants might be helpful in differentiating any cross‐reactive immunity**.** The bactericidal activity of sera from F1‐OMV immunized animals was perhaps surprising, in view of their weaker immunogenicity and the low levels of antigen‐specific antibodies they generated compared with V‐OMV vaccines (Fig. 2). The low levels of F1 expression in Bt, which required sensitive LC‐MS techniques to detect, may be a consequence of inherent differences in the translational machinery and requirements for efficient synthesis and/or in secretion sequences that were used to target newly synthesized proteins to the periplasm and OMVs in *Yersinia* *versus* *Bacteroides* species. In addition, the inability of Bt to efficiently synthesize proteins encoded within the *caf1* operon, such as *caf1M* (which encodes the CafM1 protein that acts as a chaperone for F1 with a role in its post‐translational folding and secretion 43, 44), could also compromise Bt expression of F1. The inability to detect F1 in F1‐OMV lysates using various antibodies in immunoblotting protocols may also be indicative of low levels of expression. Alternatively, it could be that the protein was not being expressed in its native form, or that expression of altered structural determinants resulted in the loss of immune epitopes following expression in Bt and OMVs. In summary, the results from the two independent assays provide compelling evidence for the development of a protective immune response in OMV‐immunized NHPs.

Studies performed with various animal species (including NHPs 45) indicate that, although neutralizing antibodies provide protection against exposure, the development of cell‐mediated immunity is essential for protection and clearance of bacteria from the host. Studies using mice with targeted mutations that disrupt T helper type 1 (Th1) or Th2 CD4 T cells responses have shown that Th1‐driven cell‐mediated immune responses are particularly important in protecting against plague 46. The ability of the V protein to up‐regulate IL‐10 production, which down‐regulates the generation of proinflammatory cytokines such as TNF‐α and IFN‐γ, is a key mechanism for virulence and immunosuppression; this contributes to the disruption of a balanced Th1/Th2 response which, alongside specific antibodies, appears to be optimal for protection 3. In this context the recall response of lymphocytes from V‐OMV immunized animals, which is characterized by the secretion of various proinflammatory cytokines, is significant and of predicted benefit to the mobilizing (MCP‐1, IL‐8) and activating (IL‐1β, IL‐6, IL‐23) components of cell‐mediated immune responses in response to plague infection in immunized animals. Of note, the lowest dose (12·5 μg) of intranasally administered V‐OMVs was particularly effective at eliciting cytokine secretion by PBMCs (Fig. 5). The reasons for this phenomenon are unclear, but it is possible that higher doses of V‐OMVs may have elicited qualitatively different (tolerogenic) immune responses similar to that described for other nanoparticle‐based vaccine delivery systems 47.

In summary, the key findings from our study were that Bt OMVs could express plague antigens, and in particular the V antigen, in a stable and correct immunogenic form. These engineered OMV vaccine formulations elicited specific immune and antibody responses both in the serum and at mucosal surfaces, including the generation of antibodies able to kill plague bacteria. Our results also highlight the key advantages our Bt OMV vaccine technology offers over available plague vaccines in terms of technology and approach. First, OMV vaccine delivery via oral or nasal administration allows for needle‐free, multi‐dose delivery that would enable mass vaccination programmes in challenging environments and at relatively low cost. Advantageously, this route of immunization also specifically targets the primary sites of mucosal infection which injectable whole‐cell vaccines or subunit vaccines do not. Secondly, compared with subunit or whole cell vaccines, the manufacture and reformulation of OMV vaccines is quicker, and can be achieved using readily accessible and relatively inexpensive technology that has been commercially validated in the production of licensed MenB OMV vaccines which are in current use 48. Thirdly, patient acceptance is expected to be high, and unlike injection‐based vaccines only require out‐of‐clinic management. Fourthly, OMVs have intrinsic adjuvanticity and the ability to activate both the innate and adaptive arms of the immune system 22, 23 compared with the requirement for chemical adjuvants such as alum to improve immunogenicity of subunit vaccines. Fifthly, OMV vaccines are acellular and non‐infectious, making them safer than live attenuated or killed whole cell vaccines. Finally, OMVs are stable for ultra‐long periods in liquid and lyophilized form 49, and for several weeks in solution form across a wide range of temperatures including 40^°^C. This allows distribution to the point of need without cold chain or cold storage, which is particularly important in tropical and low‐income settings. These properties enable Bt‐OMV vaccines to align well with the WHO Blueprint therapeutic product profile 11.

## Author contributions

Conceptualization: S. R. C., S. F., E. D. W., M. S.; investigation: A. C., A. M. C., R. S., U. W., E. J., N. J. W., S. H., M. D.; formal analysis: A. C., A. M. C., A. T., N. J. W., P. B., S. F., E. D. W., S. H., M. D.; funding acquisition: S. R. C., M. S.; project administration: M. S.; writing, original draft preparation: S. R.C.; writing, review and editing: A. M. C., R. S., E. J. , S. F., E. D. W.

## Disclosures

The authors declare no financial or commercial conflicts of interest.

## Supporting information


**Fig. S1.** Schematic of OMV plague vaccine NHP immunisations via the intranasal (IN) or oral route and analyses**.**

**Fig. S2.** Mucosal humoral immune response to F OMV vaccine (a to d). Bronchoalveolar lavage fluid (BAL) (e) and salivary gland homogenates (f) were analysed for antigen specific IgA at the study end point. The data shown represents mean ± SEM values.

**Fig. S3.** Histopathological sections of immunised macaques receiving 50 ug dose stained with H E. A. Spleen showing activated splenic follicles (f) within the white pulp. B. Lymph node showing active lymphoid follicles(f) within the cortex. C. Duodenum. Lympho plasmacytic infiltration within the mucosa and submucosa (arrow). D. Jejunum. Lympho plasmacytic infiltration within the mucosa and submucosa, showing proliferation of lymphoid follicle like structures (arrow). E. Ileum. Lympho plasmacytic infiltration within the mucosa and submucosa adjacent to activated Peyer Patches (arrow). F. Lung. Focal proliferation of the BALT without any presence of pathogen within the organ parenchyma.
