# Supplementary figures and images for: Use of bioengineered human commensal gut bacteria‐derived microvesicles for mucosal plague vaccine delivery and immunization
Source: Clin Exp Immunol. 2019 Apr 15;196(3):287–304. doi: 10.1111/cei.13301 (PMC6514708; doi:10.1111/cei.13301)

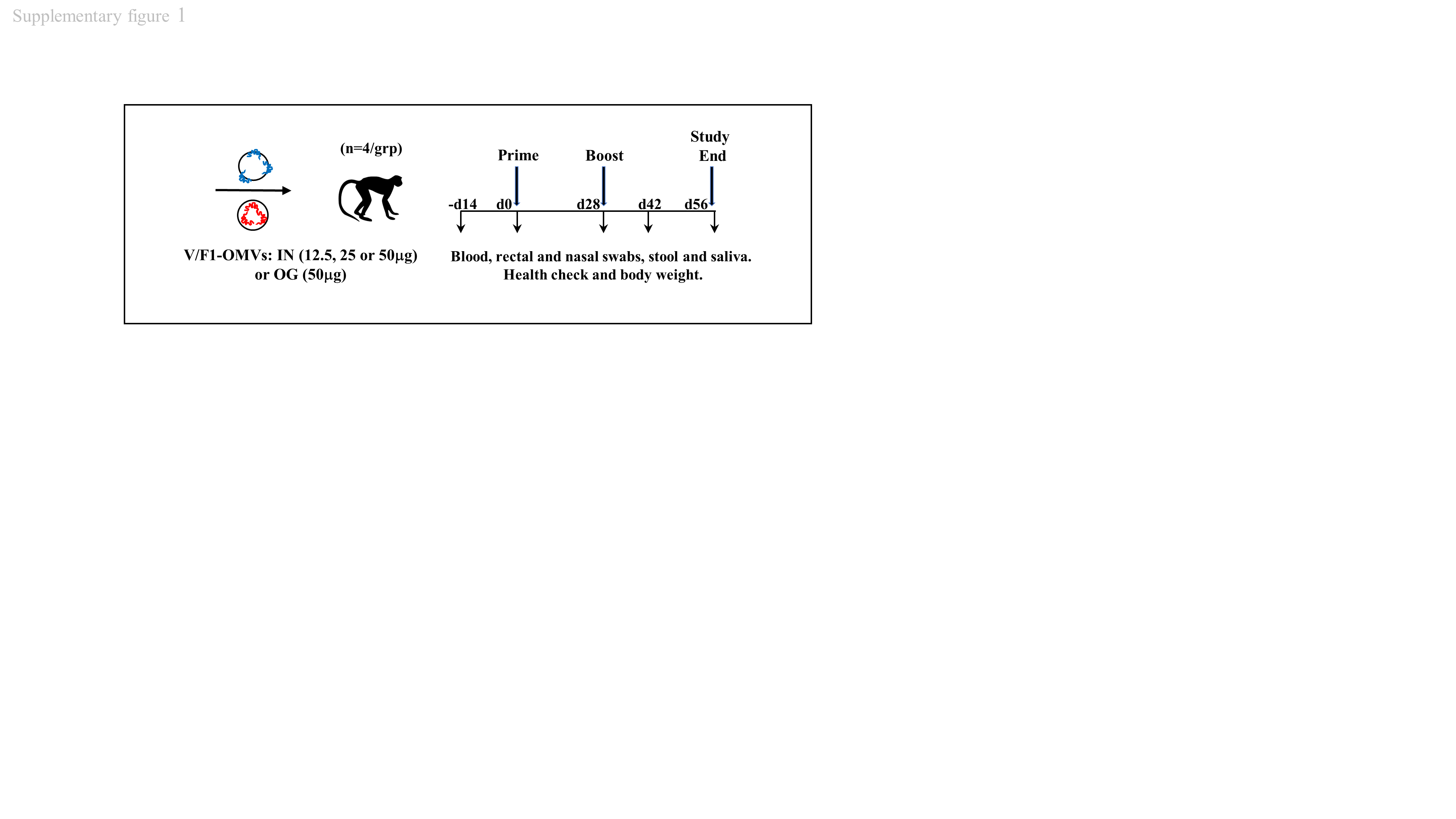

Supplement: Supplementary file 1 — Fig. S1. Schematic of OMV plague vaccine NHP immunisations via the intranasal (IN) or oral route and analyses. [file CEI-196-287-s001.tif]

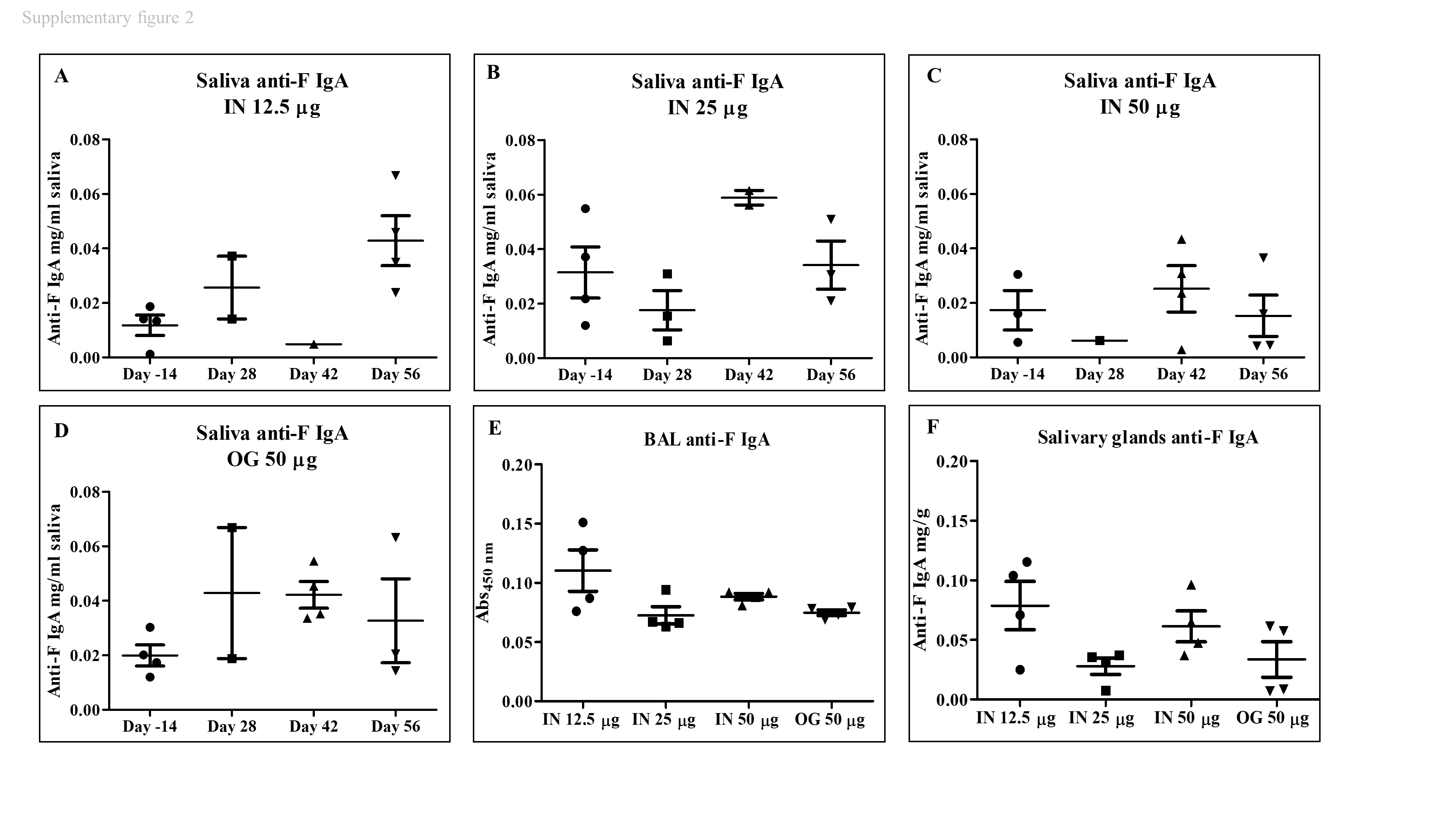

Supplement: Supplementary file 2 — Fig. S2. Mucosal humoral immune response to F OMV vaccine (a to d). Bronchoalveolar lavage fluid (BAL) (e) and salivary gland homogenates (f) were analysed for antigen specific IgA at the study end point. The data shown represents mean ± SEM values. [file CEI-196-287-s002.tif]

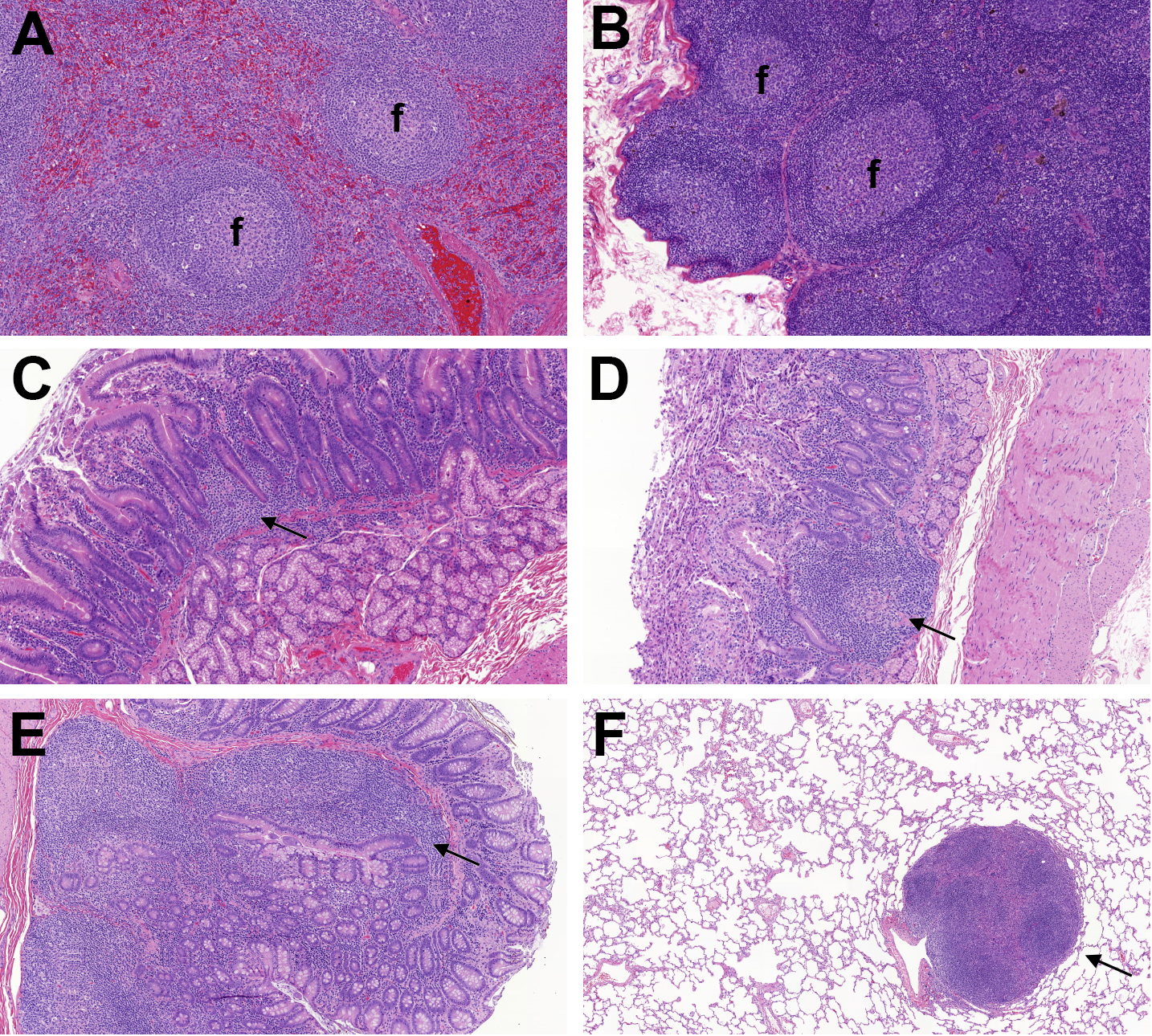

Supplement: Supplementary file 3 — Fig. S3. Histopathological sections of immunised macaques receiving 50 ug dose stained with H E. A. Spleen showing activated splenic follicles (f) within the white pulp. B. Lymph node showing active lymphoid follicles(f) within the cortex. C. Duodenum. Lympho plasmacytic infiltration within the mucosa and submucosa (arrow). D. Jejunum. Lympho plasmacytic infiltration within the mucosa and submucosa, showing proliferation of lymphoid follicle like structures (arrow). E. Ileum. Lympho plasmacytic infiltration within the mucosa and submucosa adjacent to activated Peyer Patches (arrow). F. Lung. Focal proliferation of the BALT without any presence of pathogen within the organ parenchyma. [file CEI-196-287-s003.tif]
